# The fatty acid receptor CD36 promotes HCC progression through activating Src/PI3K/AKT axis-dependent aerobic glycolysis

**DOI:** 10.1038/s41419-021-03596-w

**Published:** 2021-03-26

**Authors:** Xiaoqing Luo, Enze Zheng, Li Wei, Han Zeng, Hong Qin, Xiaoyu Zhang, Meng Liao, Lin Chen, Lei Zhao, Xiong Z. Ruan, Ping Yang, Yaxi Chen

**Affiliations:** 1 Centre for Lipid Research and Key Laboratory of Molecular Biology for Infectious Diseases (Ministry of Education), Department of Infectious Diseases, Institute for Viral Hepatitis, The Second Affiliated Hospital, Chongqing Medical University, 400016 Chongqing, China; 2 John Moorhead Research Laboratory, Centre for Nephrology, University College London Medical School, University College London, Royal Free Campus, London, NW3 2PF UK

**Keywords:** Cancer metabolism, Tumour biomarkers

## Abstract

Metabolic reprogramming is a new hallmark of cancer but it remains poorly defined in hepatocellular carcinogenesis (HCC). The fatty acid receptor CD36 is associated with both lipid and glucose metabolism in the liver. However, the role of CD36 in metabolic reprogramming in the progression of HCC still remains to be elucidated. In the present study, we found that CD36 is highly expressed in human HCC as compared with non-tumor hepatic tissue. CD36 overexpression promoted the proliferation, migration, invasion, and in vivo tumor growth of HCC cells, whereas silencing CD36 had the opposite effects. By analysis of cell metabolic phenotype, CD36 expression showed a positive association with extracellular acidification rate, a measure of glycolysis, instead of oxygen consumption rate. Further experiments verified that overexpression of CD36 resulted in increased glycolysis flux and lactic acid production. Mechanistically, CD36 induced mTOR-mediated oncogenic glycolysis via activation of Src/PI3K/AKT signaling axis. Pretreatment of HCC cells with PI3K/AKT/mTOR inhibitors largely blocked the tumor-promoting effect of CD36. Our findings suggest that CD36 exerts a stimulatory effect on HCC growth and metastasis, through mediating aerobic glycolysis by the Src/PI3K/AKT/mTOR signaling pathway.

## Introduction

Hepatocellular carcinoma (HCC) is the most popular cancer and the third leading cause of cancer-related deaths worldwide^1,2^. Metabolic reprogramming is a key feature of cancer, including HCC, which allows tumor cells to obtain the biological macromolecules needed for rapid proliferation, while meeting its own energy requirements^3^. Metabolic reprogramming involves a variety of effects and steps, but the most widely known is the Warburg effect, in which cancer cells favor the use of less efficient aerobic glycolysis rather than mitochondrial oxidative phosphorylation, to metabolize glucose for cellular proliferation^4^. Aerobic glycolysis can supplement ATP production as soon as possible, which is extremely beneficial to the rapidly proliferating tumor cells. Increasing metabolic demands make HCC tumor exhibits a high level of glucose metabolism. Glycolytic enzymes, including the hexokinase-II, glucose transporters, and pyruvate kinase M2, are highly expressed in HCC and correlated with the pathological stage of HCC tumor^5–8^. Besides, there is increasing evidence that the altered lipid metabolism pathway is associated with the pathogenesis of HCC^9^. Changes in the expression of enzymes related to fatty acid synthesis and oxidation, such as acetyl-CoA carboxylase, fatty acid synthase, and carnitine palmitoyltransferase1, are found in HCC tumors^10,11^. Nonetheless, the detailed mechanisms involved in metabolic reprogramming of HCC are still poorly understood. Hence, there is a pressing need to better understand and target the metabolism changes on pathogenesis of HCC.

Cluster of differentiation 36 (CD36) is an integral transmembrane glycoprotein expressed in various tissues, where it is involved in high-affinity uptake of long-chain fatty acids^12^. The importance of CD36 on fatty acid metabolism has been well demonstrated. In human and mice, CD36 deficiency reduces fatty acid uptake by the heart, skeletal muscle, and adipose tissue^13^. CD36 forms a complex with Fyn and serine/threonine kinase LKB1 to maintain fatty acid β-oxidation through regulating AMPK activation^14^. Our previous study showed that overexpression of CD36 facilitates fatty acid uptake and impairs fatty acid oxidation, thus leading to the progression of nonalcoholic fatty liver disease (NAFLD)^15^. On the other hand, CD36 knockdown upregulates fatty acid oxidation and lipophagy pathway, thereby reducing hepatic lipid accumulation^16^. Interestingly, emerging evidence has revealed a relationship between CD36 and glucose metabolism. Mice with CD36-depleted muscle have attenuated insulin signaling and suppressed insulin-induced glucose utilization^17^. A recent study of ours indicated that loss of CD36 impairs insulin action and increases glucose production in the liver^16,18–20^. These above data indicate a role of CD36 in both lipid and glucose homeostasis.

A possible emerging role of CD36 in cancer has been proposed recently. Notably, CD36-mediated fatty acid metabolism plays a significant role in the growth and metastasis of multiple tumors, including oral cancer, breast cancer, cervical cancer, and gastric cancer. It was reported that CD36-mediated fatty acid oxidation through an unknown mechanism is a possible initiator for oral squamous cell carcinoma metastasis^21^. As a typical fatty acid transporter, saturated and monounsaturated fatty acids promote tumor proliferation and migration via a CD36-dependent pathway^22^. Our early work showed that dietary oleic acid (18 : 1)-induced CD36 promotes cervical cancer cell growth and metastasis^23^. CD36 was also found to be involved in palmitic acid (16 : 0)-induced cell migration and invasion via the AKT/GSK-3β/β-catenin signaling pathway, thus promoting the metastasis of gastric cancer^24^. Another study towards NAFLD-associated HCC indicated that CD36-mediated oxidized low density lipoprotein (oxLDL) uptake induces its carcinogenic signaling^25^. Beyond that, regulation of CD36 expression may also involve in other processes of tumor development, such as tumor angiogenesis and tumor immunity^26^. Nevertheless, glucose metabolism signaling pathways in HCC regulated by CD36 have been poorly explored.

In the present work, we attempted to investigate the role of CD36 in HCC progression and explore the potential molecular mechanisms. We found that CD36 is highly expressed in HCC and elevated CD36 expression contributes to tumor growth and metastasis in vitro and in vivo. Metabolic phenotype analysis revealed CD36-overexpressed HCC cells have a stronger glycolytic potential rather than oxidative phosphorylation. Furthermore, we explored the downstream signaling pathways by which CD36 mediates glycolysis and tumor growth. Based on this study, we found CD36 exerts a novel role in mediating oncogenic glycolysis and suggest that it might be a therapeutic target for HCC.

## Materials and methods

### Human tissue samples

HCC tissues and the adjacent noncancerous tissues (*n* = 30) were collected from patients who were identified using the Barcelona Clinic Liver Cancer guidelines definition and underwent surgical treatment at the Second Affiliated Hospital of Chongqing Medical University (Chongqing, China) between 2016 and 2019; all patients provided informed consent. All specimens were obtained immediately after surgical resection, snap frozen in liquid nitrogen, and kept at −80 °C. The study was approved by the Committee on Ethics of the Second Affiliated Hospital of Chongqing Medical University.

### Animal models

Six- to 8-week-old, male BALB/c-nu nude mice (*n* = 9 for each group) were obtained from the Animal Experimental Center of Chongqing Medical University (Chongqing, China). No randomization was used when animals were selected. All animal experiments were approved by the Institutional Animal Care and met the standards set by the Ethics Committee of Experiment Animals. Animals were fed in air-conditioned room with abundant water and oxygen. Animals were randomly divided into two groups and then HCC cells with or without CD36 overexpression (1 × 10^6^) were injected subcutaneously in their left flanks. The weight of the mice was recorded every 7 days, the length and width of the subcutaneous xenograft tumors were recorded every 3 days, and the volume was calculated as described previously^27^.

### Cell culture

Immortalized normal hepatocyte L02 and five types of HCC cells, named Huh7, SK-hep-1, SMMC-7721, HepG2, PLC/PRF/5, were cultured in high glucose Dulbecco’s modified Eagle medium (DMEM) containing 10% fetal bovine serum (FBS), 100 U/ml penicillin and 100 U/ml streptomycin at 37 °C under 5% CO_2_ atmosphere. These cell lines were authenticated by Short Tandem Repeat (STR) profiling and not contaminated by mycoplasma. The lentivirus containing CD36 cDNA were synthesized to create a CD36-overexpressed (CD36 OE) stable cell line and an empty vector was used as the control. The same protocol was used to establish a CD36-knockdown (CD36 RNAi) cell line. Puromycin was used to select all transfected cells. Shanghai Genechem Co., Ltd (Shanghai, China) was the place where we purchased all lentiviruses.

### Cell proliferation assay

To investigate the effect of CD36 OE or shCD36 on proliferation of HCC cells, after grouping, the cells were seeded in a 96-well plate with 3000 cells per well. Long time cell dynamic imaging system (IncuCyte ZOOM, Essen BioScience, USA), cell counting kit-8 (CCK-8) assay, and 5-Ethynyl-2’-deoxyuridine (EdU) assay were used to assess the capability of cell proliferation. In CCK-8 assay, the optical density (OD) values were measured at 450 nm after incubation with CCK-8 reagent for 2 h at 37 °C. EdU assay was performed according to the manufacturer’s protocols.

### Cell cycle analysis

The cells at logarithmic growth stage were inoculated in a six-well plate. The cells were collected after 48 h, the cell density was adjusted to 1 million per milliliter, and was fixed by pre-cooled anhydrous ethanol. Cell cycle was measured by flow cytometry.

### Bioenergetics assay

Cell energy metabolism phenotype and glycolytic capacity were measured by the Seahorse XFe 24 Analyzer (Agilent, USA). The night before the experiment, the cells (2 × 10^4^) were planted in a 24-well plate. The sensor cartridge was calibrated with XF Calibrant and placed in a non-CO_2_ 37 °C incubator overnight. On the day of the assay, the growth medium was replaced by assay media. In the energy phenotype experiment, the assay media were seahorse XF Base medium containing 1 mM pyruvate, 2 mM glutamine, and 10 mM glucose. Carbonyl cyanide 4-(trifluoromethoxy) phenylhydrazone (FCCP) and oligomycin (Sigma, USA) were dissolved in the assay medium and stressor mix was created; 55 μl of the stressor mix (the final concentration of both is 1 μM) was loaded into each port A of a hydrated sensor cartridge. The assay media of glycolytic capacity consisted of 2 mM glutamine in seahorse XF Base medium. Fifty-six microliters of glucose (10 mM final concentration), 62 μl of oligomycin (3 μM final concentration), and 69 μl of 2-Dexoy-D-Glucose (2-DG) (50 mM final concentration) were added to the appropriate injection ports.

### Transwell assays

The transwell assays were divided into transwell migration and transwell invasion assay. The basic operation was as follows: the transwell chamber was placed into the 24-well culture plate, the chamber was called the upper chamber, and the culture plate was called the lower chamber. The cells were resupended in serum-free medium with or without inhibitors (phosphatidylinositol 3-kinase (PI3K) inhibitor: BKM-120; AKT inhibitor: MK-2206; mammalian target of rapamycin (mTOR) inhibitor: rapamycin) and were seeded in the upper chamber; DMEM containing 10% FBS is generally added to the lower chamber. Forty microliters of matrigel (BD Biosciences, USA) was used to coat the upper membrane in advance for the transwell invasion assay, the cells were fixed with 4% paraformaldehyde, and stained with 0.1% crystal violet (Solarbio, China) after 24 h of migration experiment or 48 h of invasion experiment.

### Wound healing

The cells were inoculated in a six-well plate and the monolayer was scratched with a pipette tip when the cells were up to 90% density; the cells were treated with or without inhibitor (the types of inhibitors are as described above). ImageJ software was used to calculate the wound areas.

### Histology and immunohistochemistry analysis

The protocol of hematoxylin and eosin (HE) staining and immunohistochemistry (IHC) analysis have been introduced in detail before. The following antibodies were used: anti-CD36 (1 : 800, Novus) and anti-PCNA (1 : 8000, CST).

### Real-time quantitative PCR

TRIzol reagent (Takara; China) was used to extract total RNA. The RNA was purified and reverse-transcribed into cDNA using a reverse transcription kit (Takara, China). The cDNA products were amplified according to a two-step PCR method. The primers of CD36 and β-actin were synthesized by Tsingke Biological (Chongqing, China). Relative mRNA levels of CD36 were calculated using the 2^−ΔΔCt^ method. The internal reference gene was β-actin.

### Western blot analysis

Protocol used for western blotting has been described previously^28^. The following dilutions of antibodies were used: anti-CD36 (1:2000, Novus, Cat# NB400-144), anti-AKT(1:1000, CST, Cat#4691T), anti-p-AKT (1:2000, CST, Cat#4060S), anti-mTOR/anti-p-mTOR (1:1000, CST, Cat#2983S/5536S), anti-PI3K(1:1000, CST, Cat#4257T), anti-p-PI3K (1:500, GeneTex, Cat# GTX132597), anti-Src (1:1000; CST, Cat#2109T), anti-p-Src(1:1000; CST, Cat#6943T), and anti-β-actin (1:5000, Bioss, Cat# bs-0061R). The relative protein levels were semi-quantified by ImageJ software.

### Statistical analysis

The two-tailed unpaired Student’s *t*-test was used to compare the difference of two groups and one-way analysis of variance with Tukey’s multiple comparison tests was used when more than two groups were compared. There were no studies in which investigators were blinded and all experiments were repeated at least three times. No statistical method was used to predetermine sample size. The sample size was chosen on the basis of literature in the field. All data were presented as the mean ± SD and *P* < 0.05 was considered significant.

## Results

### The expression of CD36 is upregulated in HCC

**Fig. 1:**
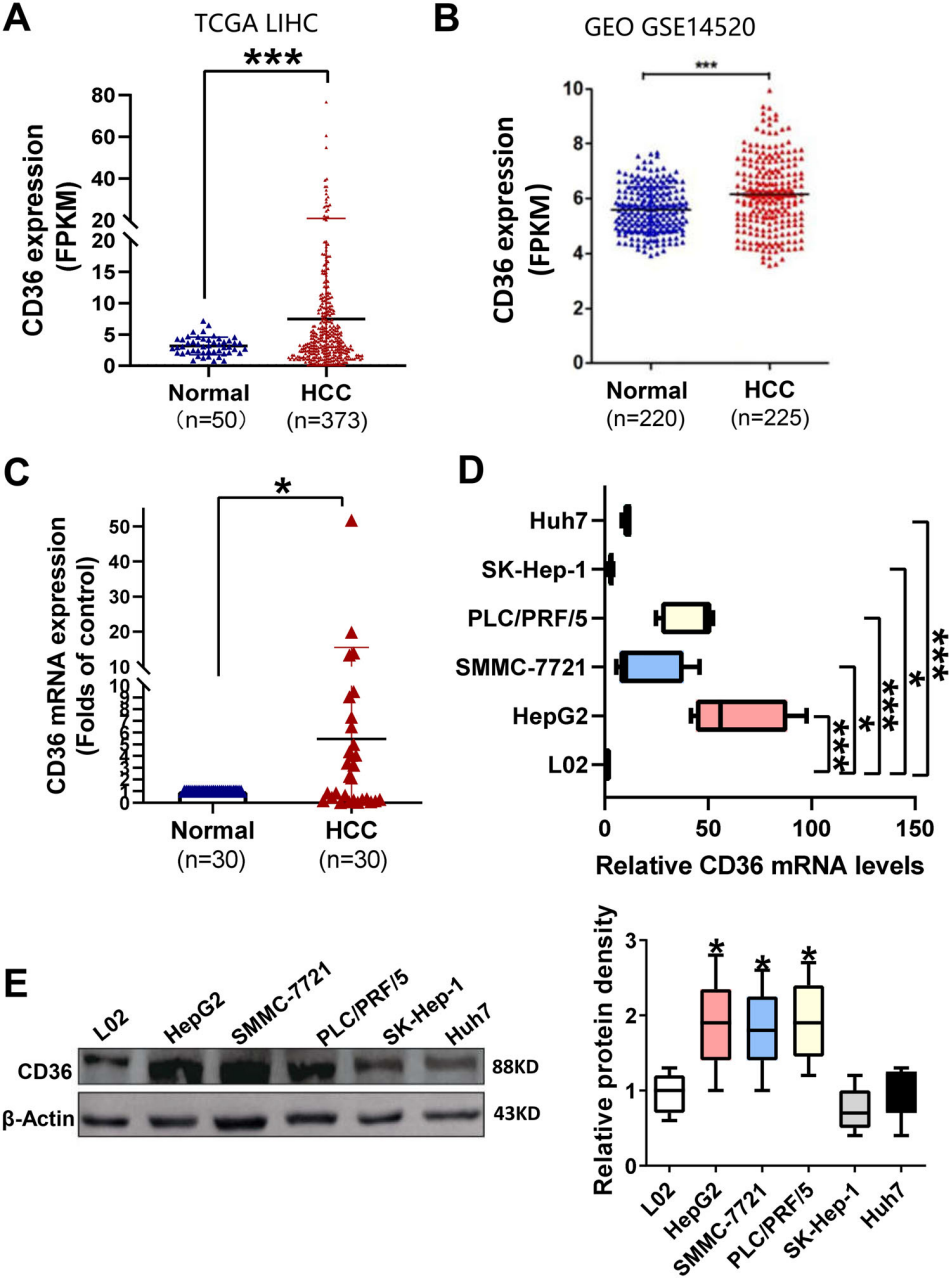
CD36 levels are increased in human HCC samples and cell lines. The expression of CD36 in 373 HCC samples (including 50 paired normal liver tissues) from TCGA dataset (**A**) and 225 HCC samples (including 220 paired normal liver tissues) from GSE14520 dataset (**B**) was analyzed. **C** The mRNA levels of CD36 in 30 paired HCC and their paracancerous tissues were examined by RT-qPCR. *P < 0.05, **p < 0.01, and ***p < 0.01 compared with the normal tissue. The mRNA and protein levels of CD36 in HCC cell lines, named L02, Huh7, SK-hep-1, SMMC-7721, HepG2, and PLC/PRF/5, were measured by real-time PCR (**D**, *n* = 5) or western blotting (**E**, *n* = 3). The densitometric quantification of the blots with all cell lines was shown in the following. All data are presented as mean ± SD. *P < 0.05, **p < 0.01, and ***p < 0.001 compared with the L02 cell line. *P*-value was calculated using Student’s *t*-test.

To evaluate the potential role of CD36 in HCC pathogenesis, we analyzed CD36 expression in human HCC tissues from The Cancer Genome Atlas (TCGA) dataset. The mRNA expression of CD36 in HCC tissues (*n* = 373) was higher than that of in paired normal liver tissues (*n* = 50) according to the TCGA dataset (Fig. 1A). Consistently, GSE14520 dataset (including 225 HCC samples and 220 paired normal samples) from the Gene Expression Omnibus revealed that CD36 is increased in HCC tissues (Fig. 1B). Furthermore, we examined CD36 expression in 30 paired HCC and their paracancerous tissues by reverse-transcriptase quantitative PCR. The mRNA levels of CD36 were significantly elevated in HCC tissues than in corresponding noncancerous tissues (Fig. 1C).

Then, the mRNA and protein levels of CD36 were analyzed in five types of HCC cell lines, including HepG2, SMMC-7721, SK-Hep-1, Huh7, and PLC/PRF/5, using normal L02 hepatocyte as control. The results showed that the expression of CD36 is abundant in SMMC-7721, HepG2, and PLC/PRF/5 cell lines at both the mRNA and protein levels compared with L02. Despite the CD36 mRNA levels were elevated by several folds in SK-Hep-1 and Huh7 cell lines, there was no apparent difference at protein levels when compared with L02 cells (Fig. 1D, E).

### CD36 knockdown suppresses the proliferation and migration of HCC cells

**Fig. 2:**
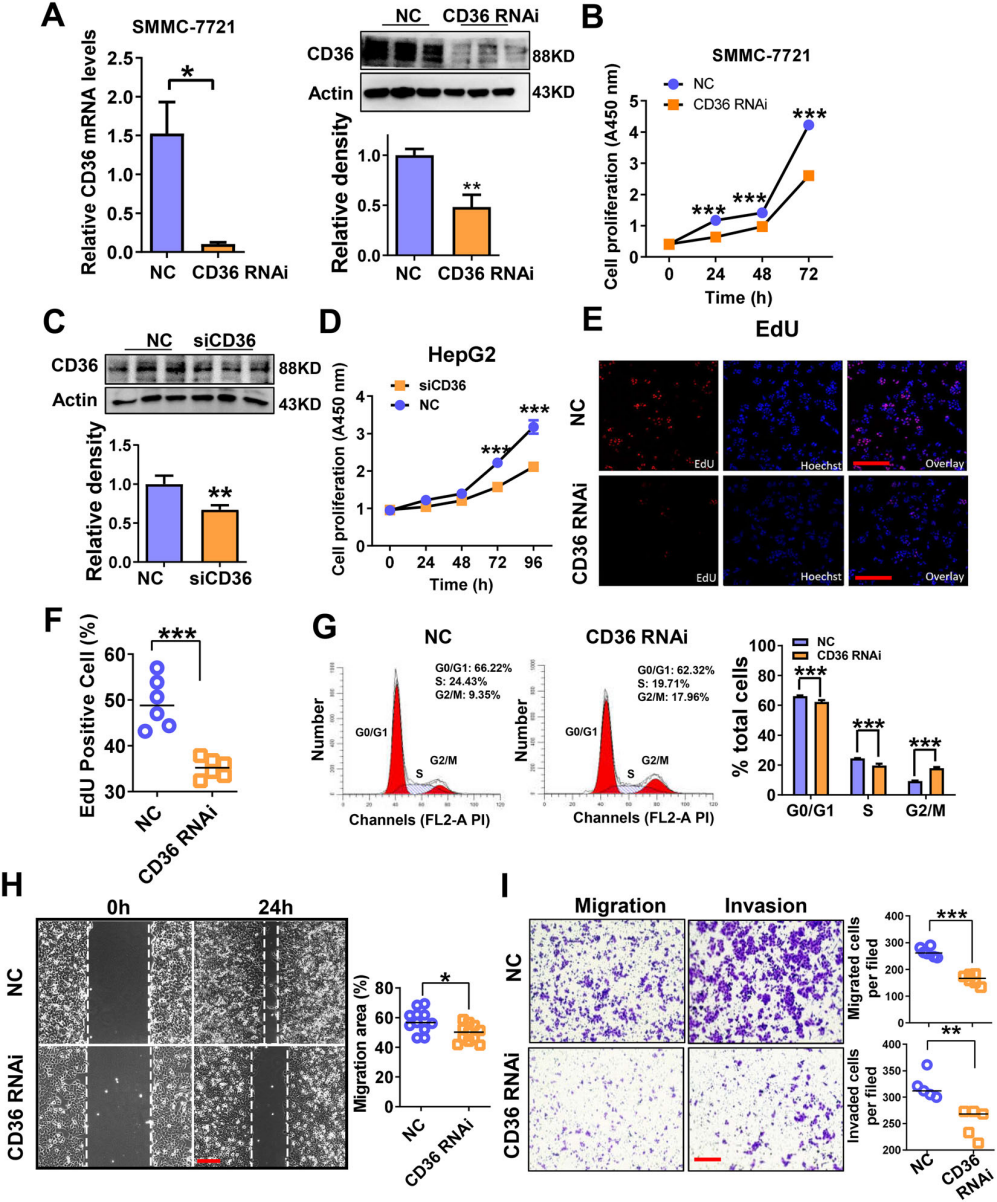
CD36 knockdown has the negative impact on the proliferation, migration, and invasion of HCC cells. CD36-knockdown (CD36 RNAi) cell line was established by transfecting CD36 shRNA lentiviral construct and targeting sequence 5′-GGCTGTGTTTGGAGGTATT CT-3′ or scrambled shRNA lentivirus as control (NC) to SMMC-7721 cells. **A** The knockdown efficiency of CD36 in SMMC-7721 cells was detected by real-time PCR and western blotting (*n* = 3). **B** The proliferation of the control and CD36 RNAi cells in SMMC-7721 cells was measured by CCK-8 assay (*n* ≥ 5). **C** The knockdown efficiency of CD36 in HepG2 cells was detected by western blotting (*n* = 3). **D** The proliferation of the control and CD36 RNAi cells in HepG2 cells was measured by CCK-8 assay (*n* ≥ 5). **E** The proliferation of the control and CD36 RNAi cells in SMMC-7721 cells was measured by EdU assay, the Edu-positive cells were shown in the following (**F**) (*n* ≥ 5). **G** Cell cycle analysis was performed after culture for 48 h using flow cytometer (*n* = 3). **H** Scratch-wound cell migration assay of the control and CD36 RNAi cells (*n* = 5). **I** Cell migration and invasion ability was evaluated by transwell assay (*n* = 5). Cell number refers to the average number ± SEM per field counted at ×200 magnification. *P < 0.05, **p < 0.01, and ***p < 0.001 compared with the NC group. Bar = 100 μm (**C**, **E**, **F**). *P*-value was calculated using Student’s *t*-test.

As CD36 expression is abundant in SMMC-7721, HepG2, and PLC/PRF/5 cell lines, therefore, the effects of CD36 knockdown on cell proliferation, migration, and invasion were evaluated in these cell lines. Short hairpin RNA lentiviral transfection was used to silence CD36 expression in SMMC-7721 cells and the knockdown efficiency was validated by reverse-transcriptase PCR and western blotting (Fig. 2A). CCK-8 assay showed that CD36 knockdown impairs the proliferation of SMMC-7721 cells (Fig. 2B). In addition, in HepG2 and PLC/PRF/5 cell lines, small interfering RNA-mediated knockdown of CD36 also inhibited cell proliferation (Fig. 2C, D and Supplementary Fig. 1A, B).

By using EdU assay, which reflects DNA synthesis and cell proliferation, we found that the number of EdU-positive cells was obviously decreased in the shCD36 group compared with that in the control group (Fig. 2E, F). In addition, cell cycle analysis showed that the percentage of cells in S and G0/G1 phase was reduced, and that of cells in G2 phase was increased by CD36 knockdown (Fig. 2G). Next, we evaluated the impact of CD36 suppression on the migration and invasion of HCC cells. Wound-healing and transwell assays showed that inhibition of CD36 expression decreased the migration and invasion ability of SMMC-7721 cells (Fig. 2H, I). These data demonstrated that CD36 silencing exerts anti-cancer effect in HCC cells.

### CD36 overexpression promotes HCC tumor growth and metastasis in vitro and in vivo

**Fig. 3:**
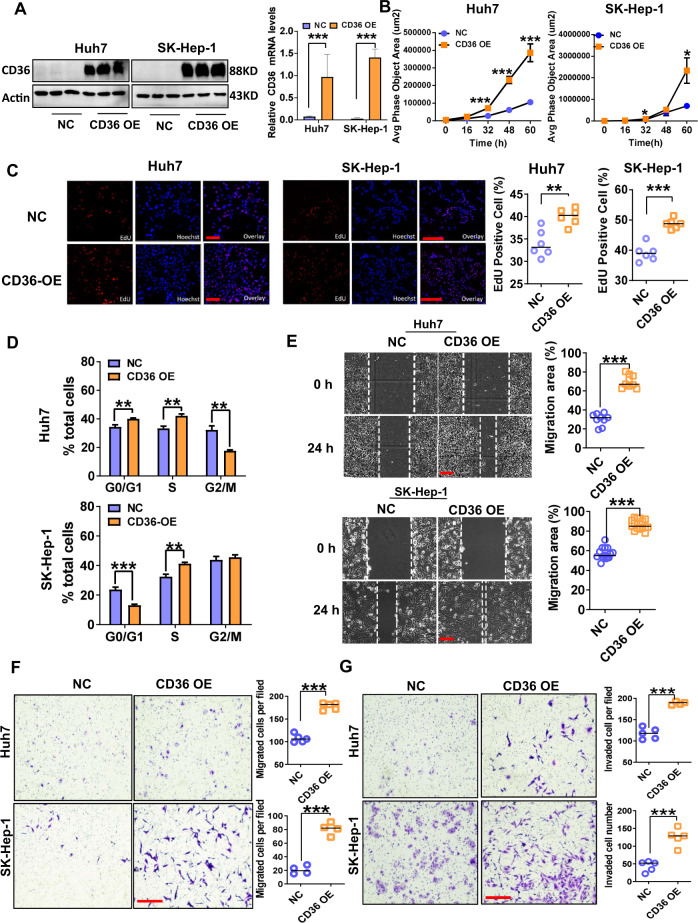
CD36 overexpression has a positive impact on proliferation, migration, and invasion of HCC cells. CD36-overexpressed (CD36 OE) Huh7 and SK-Hep-1 cells were constructed by transfection of a recombinant lentivirus (Ubi-MCS-3FLAG-SV40-puromycin) containing CD36 cDNA or an empty vector as NC. **A** Western blotting analysis of CD36 levels in indicated HCC cells, actin was used as the control, the relative quantification of the blots was shown on the right (*n* = 3). The proliferation of CD36 OE cells or control cells was measured by long time cell dynamic imaging system (**B**) and EdU assay (**C**) (*n* ≥ 5). **D** Cell cycle analysis was performed after culture for 48 h using flow cytometer (*n* = 3). **E** Scratch-wound cell migration assay of CD36 OE or control cells (*n* = 5). Cell migration (**F**) and invasion (**G**) ability was evaluated by transwell assays (*n* = 5). All data are presented as mean ± SD. *P < 0.05, **p < 0.01, and ***p < 0.001 compared with the NC group. Bar = 100 μm (**C**, **E**–**G**). *P*-value was calculated using Student’s *t*-test.

CD36 expression was relatively low in SK-Hep-1 and Huh7 cell lines, so CD36 was overexpressed stably by lentiviral transfection into the two cell lines, which was confirmed by western blot analysis (Fig. 3A). Cell viability and proliferation were analyzed using a long time cell dynamic imaging system, EdU assay, and cell cycle assay. Lentiviral-mediated CD36 overexpression significantly promoted the proliferation of both SK-Hep-1 and Huh7 cells (Fig. 3B, C). At the same time, flow cytometry analysis suggested that when CD36 was overexpressed, the percentage of cells in S phase was increased (Fig. 3D). In addition, CD36 overexpression significantly accelerated HCC cell migration and invasion as detected by wound-healing and transwell assays (Fig. 3E–G).

**Fig. 4:**
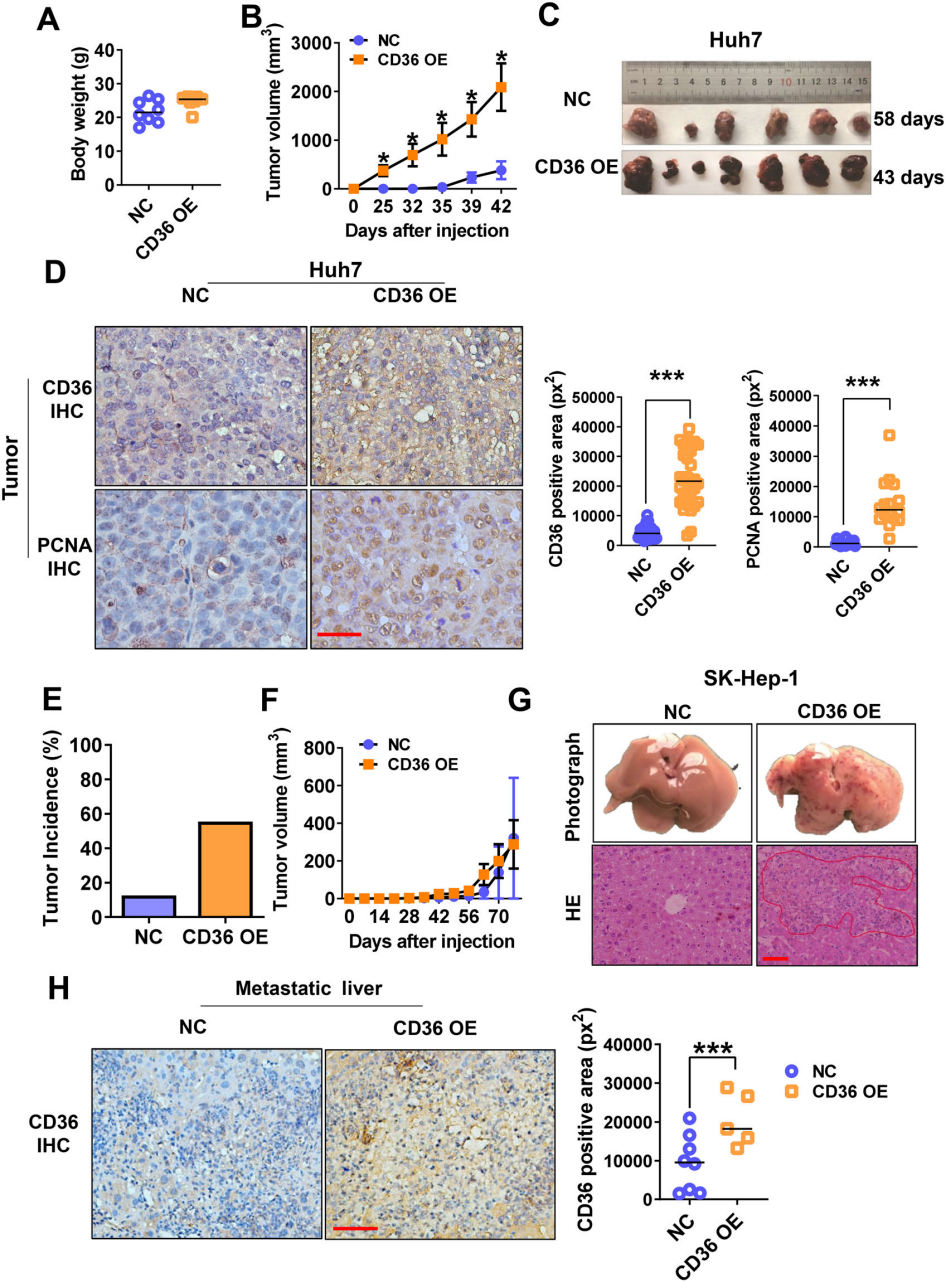
CD36 overexpression aggravates HCC growth and metastasis in mouse xenograft model. CD36 OE or control cells (1 × 10^6^) were subcutaneously injected into the left flanks of nude mice (*n* = 9). The body weight (**A**) and tumor volumes (**B**) of mice injected with Huh7 cells. **C** Photographs of subcutaneous Huh7 tumors were shown. **D** IHC analysis of PCNA and CD36 in Huh7 tumors, and the quantification of CD36-positive and PCNA-positive area was shown on the right. Representative images were shown at ×400 magnification. Tumor incidence (**E**) and tumor volumes (**F**) of mice injected with SK-Hep-1 cells. **G** The above images were representative images of liver metastases in each group after 70 days. The following images were representative images of HE staining of the metastasized liver sections. Representative images were shown at ×400 magnification. **H** IHC analysis of CD36 in SK-Hep-1 liver tissue from each group, the quantification of CD36-positive area was shown on the right. Representative images were shown at ×400 magnification. All data are presented as mean ± SD. *P < 0.05 and ***p < 0.001 compared with the NC group. Bar = 50 μm (**D**, **G**, **H**). *P*-value was calculated using Student’s *t*-test.

To determine the effects of CD36 in vivo, overexpressed and control cells were subcutaneously injected into nude mice. In the subcutaneous xenograft tumor model constructed by the Huh7 cell line, although there was no significant difference in body weight, we observed that the volume of xenograft tumors in the CD36 overexpression group were significantly larger than that of the control group (Fig. 4A, B). The tumors from the two groups were collected at different time, when they reached approximately the same size (2000 mm^3^) (Fig. 4C). IHC results showed that CD36-positive tumor cells are accompanied by higher PCNA-nuclear expression (Fig. 4D). The results of subcutaneous tumors established by SK-Hep-1 were slightly different from that of Huh7. The tumor incidence in CD36 overexpression group (five in nine mice) was higher than the control group (one in eight mice), but we did not observe the evident of tumor volume variation between the two groups of SK-Hep-1 tumors, probably because of the low number of the control tumors (Fig. 4E, F). Interestingly, CD36 OE SK-Hep-1 cells showed the presence of liver metastasis. HE staining showed that the liver metastatic nodules in the CD36 overexpression group were significantly increased, which consisted of more CD36-positive cells (Fig. 4G, H). These experimental data confirmed the role of CD36 in HCC tumor progression in vivo.

### CD36 mediates the glycolytic pathway of HCC cells

**Fig. 5:**
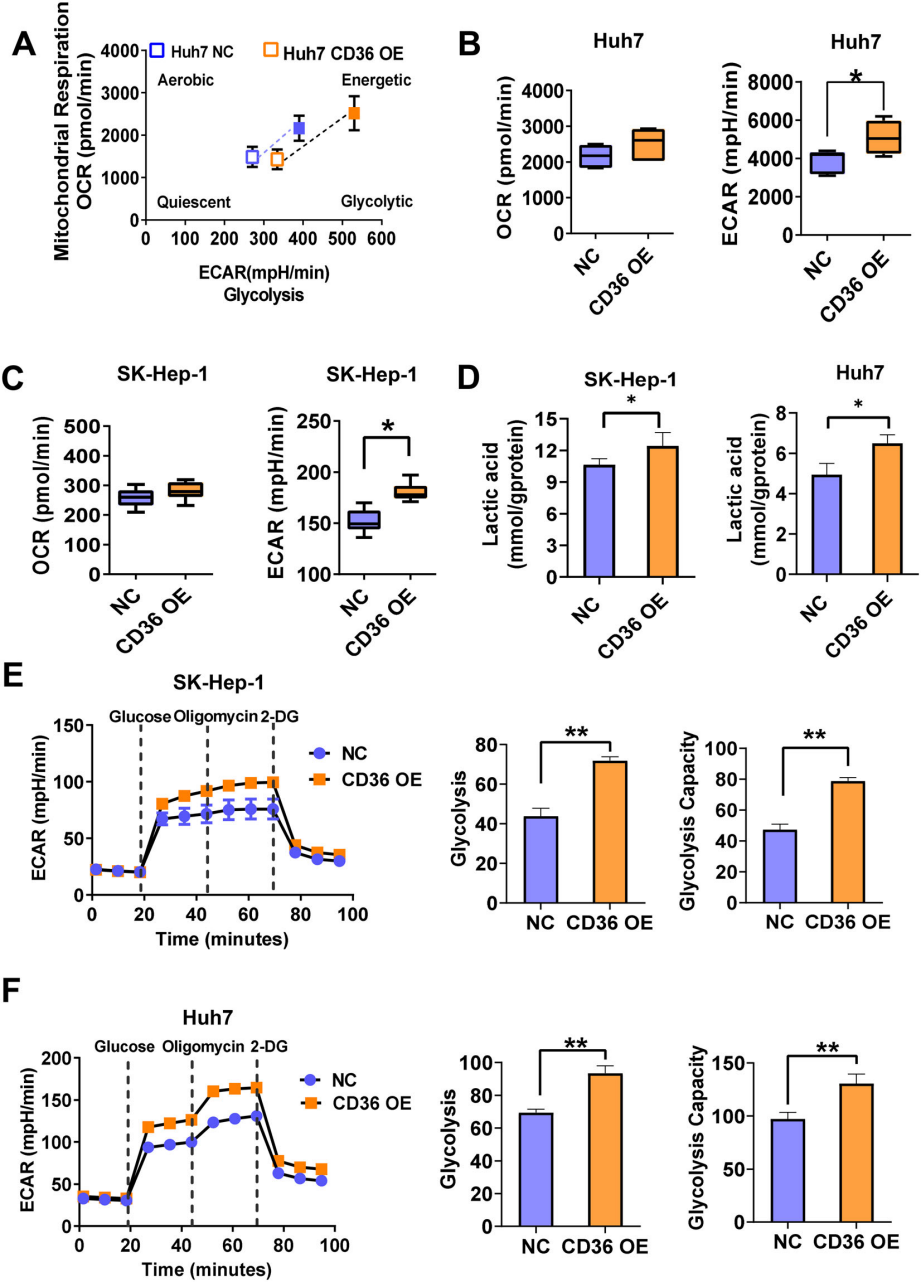
CD36 promotes glycolysis in HCC cells. The energy metabolism phenotype and glycolytic capacity of CD36 OE or control cells were measured by the Seahorse XFe 24 Analyzer. **A** Energy metabolism phenotype of CD36 OE or control Huh7 cells. **B**, **C** The stressed OCR and ECAR of Huh7 and SK-Hep-1 cells (*n* = 3). **D** The lactic acid production of SK-Hep-1 and Huh7 cells (*n* = 6). **E**, **F** Glycolytic capacity of Huh7 and SK-Hep-1 cells was measured by glycolytic stress test (*n* = 3), the key parameters of glycolytic flux including glycolysis and glycolytic capacity were shown on the right. All data are presented as mean ± SD. *P < 0.05 and **p < 0.01 compared with the NC group. *P*-value was calculated using Student’s *t*-test.

Metabolic reprogramming has been recognized as a hallmark of cancer, wherein changes of glucose and fatty acid metabolic pathways were always involved. CD36 mediated fatty acid uptake and oxidation in tumor cells, and also regulated glucose metabolism in the liver and muscle as reported by ours and others studies^17,29^. However, whether CD36 reprograms glucose metabolism of cancer cells is largely unknown. So, we used Seahorse XF analyzer (with the Cell Energy Phenotype kit) to simultaneously measure metabolic phenotypes and metabolic potential of HCC cells. In general, oxygen consumption rate (OCR) is a measure of the rate of mitochondrial respiration and extracellular acidification rate (ECAR) is a measure of the rate of glycolysis. HCC cells were prone to the glycolytic pathway when CD36 was overexpressed, showing no significant difference in OCR values but significant increase in ECAR values in CD36 OE HCC cells (Fig. 5A–C). In accordance, CD36 overexpression increased the production of lactic acid both in SK-Hep-1 and in Huh7 cells (Fig. 5D). Furthermore, the glycolytic stress test showed that CD36 overexpression boosts glycolysis and glycolytic capacity (Fig. 5E, F).

Subsequently, we evaluated the possible involvement of glycolytic pathway in CD36-mediated HCC cell proliferation and migration. The inhibitors of hexokinase (a key enzyme in the glycolytic pathway), named 2-Deoxyglucose and 3-Bromopyruvate, were then used to block glycolysis. CCK-8 assays showed that the pro-proliferation effect caused by CD36 overexpression disappeared after inhibiting the activity of hexokinase (Supplementary Fig. 1C, D). Meanwhile, both 2-Deoxyglucose and 3-Bromopyruvate decreased the migration ability induced by CD36 overexpression (Supplementary Fig. 1E–G). These results indicate that the tumor-stimulating effects of CD36 may act through CD36-mediated glycolysis.

### The mTOR pathway is involved in the regulation of glycolysis by CD36

**Fig. 6:**
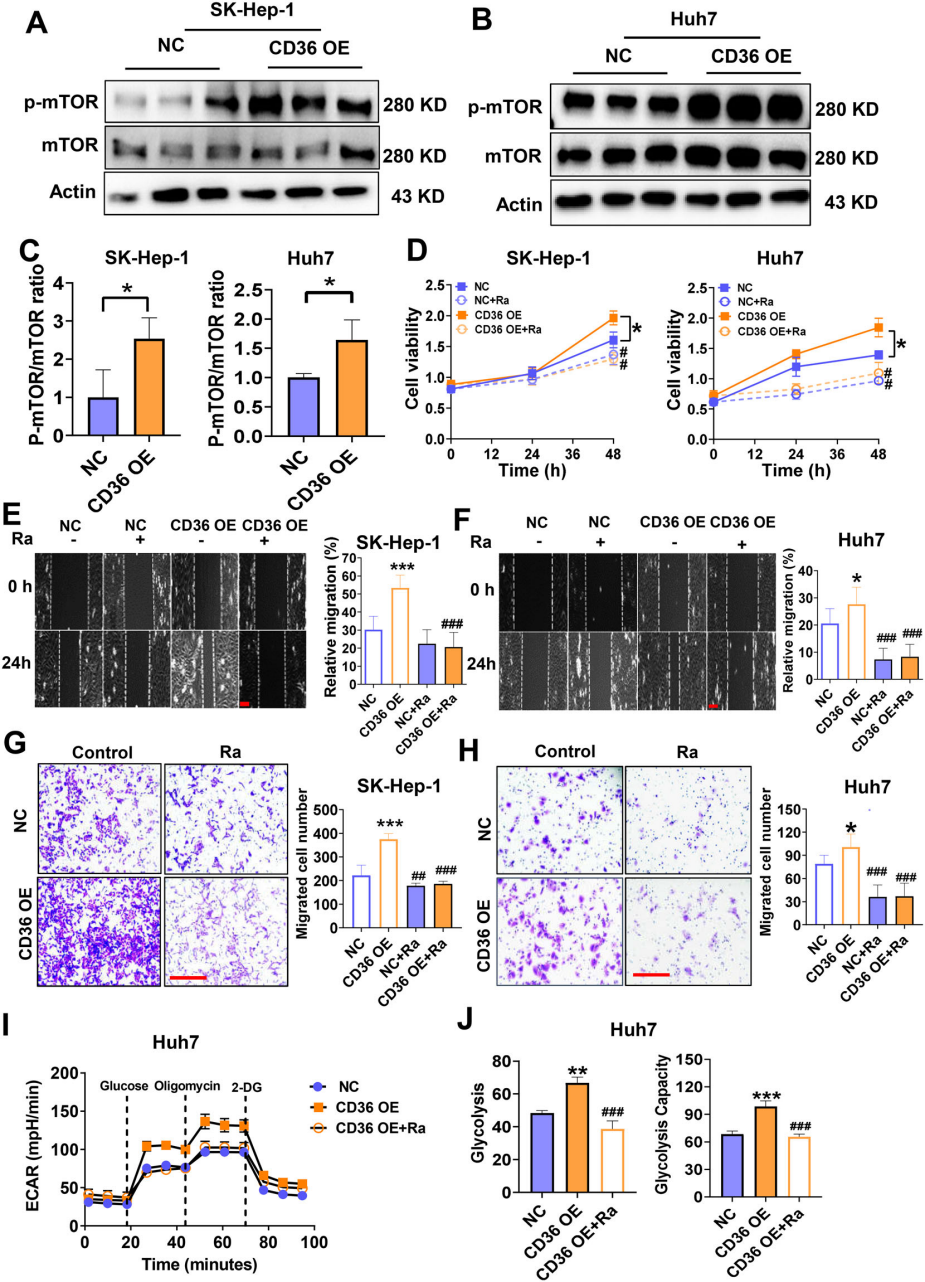
CD36 regulates glycolysis by mTOR pathway. Western blot analysis of mTOR and p-mTOR expression both in SK-Hep-1 (**A**) and Huh7 cells (**B**) (*n* = 3). **C** The histogram represented the densitometric scans for protein bands from **A** and **B**. **D** Proliferation of HCC cells treated with or without rapamycin (RA, 8 μM), an inhibitor of mTOR (n = 6). Wound-healing (**E**, **F**) and transwell assays (**G**, **H**) were used to evaluate the migration of HCC cells with or without RA (*n* = 10). **I**, **J** Glycolytic capacity of Huh7 cells with or without RA was measured by glycolytic stress test. All data are presented as mean ± SD. *P < 0.05, **p < 0.01, and ***p < 0.001 compared with the NC group, ^##^*P* < 0.01 and ^###^*p* < 0.001 compared with the group without RA. Bar = 100 μm (**E**–**H**). *P*-value was calculated using Student’s *t*-test (**C**) and one-way analysis of variance with Tukey’s multiple comparison tests (**D**–**J**).

mTOR is an important regulator of cell metabolism. Studies have demonstrated that mTOR-mediated glycolysis plays an important role in the tumor cell proliferation and migration^30,31^. Therefore, we suspect that mTOR may be the hinge that links CD36 to glycolysis. This hypothesis was then verified via western blot analysis, in which CD36 overexpression induced mTOR phosphorylation both in Huh7 and SK-Hep-1 cell lines (Fig. 6A–C). On the contrary, inhibition of CD36 effectively prevented mTOR signal (Supplementary Fig. 2A–I). We then incubated the cells with rapamycin, a pharmacological inhibitor of mTOR, to test whether mTOR pathway participates in CD36-mediated glycolysis and carcinogenesis. The finding was that rapamycin not only inhibits the proliferation of HCC cells but also eliminates the proliferative effect caused by CD36 overexpression (Fig. 6D). Moreover, pretreatment with rapamycin reversed CD36-induced cell migration and invasion as well (Fig. 6E–H). In addition, the glycolytic stress test suggested that rapamycin pretreatment eliminates the effects of CD36 overexpression on glycolysis and glycolysis capacity (Fig. 6I, J). These results indicate that mTOR is the vital molecule to connect CD36 and glycolysis.

### CD36 activates the mTOR pathway through the Src/PI3K/AKT cascade

**Fig. 7:**
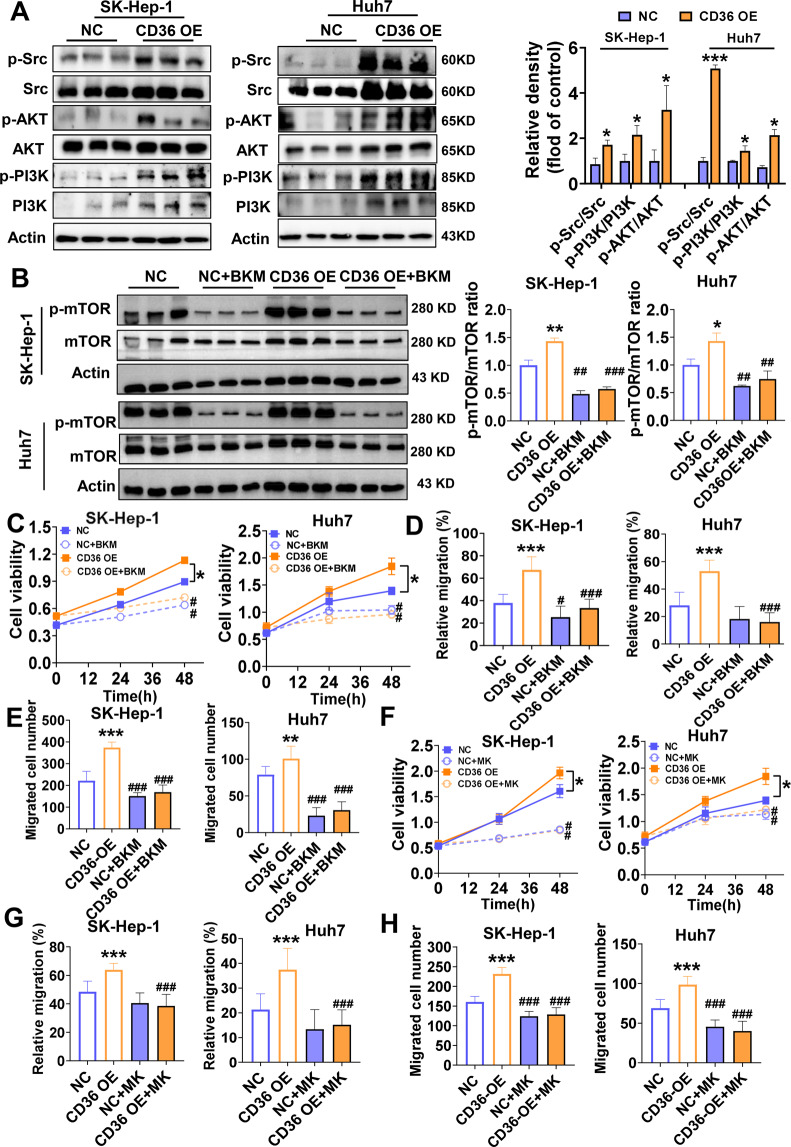
Src/PI3K/AKT is a key pathway by which CD36 activates mTOR. **A** Western blot analysis of Src, p-Src (tyr416), AKT, p-AKT (Ser43), PI3K, and p-PI3K expression both in SK-Hep-1 and Huh7 cells (*n* = 3). The relative quantification of the target proteins was shown on the right. **B** SK-Hep-1 and Huh7 cells were pretreated with BKM-120 (BKM, 1 μM) before analyzing mTOR signaling pathway (*n* = 3). The ratio of p-mTOR/mTOR was shown on the right. **C** Proliferation of HCC cells treated with or without BKM-120 (1 μM), an inhibitor of PI3K (*n* = 6). Wound-healing (**D**) and transwell assays (**E**) were used to evaluate the migration of HCC cells with or without BKM (*n* = 10). **F** Proliferation of HCC cells treated with or without MK-2206 (MK, 5 μM), an inhibitor of AKT (*n* = 6). Wound-healing (**G**) and transwell assays (**H**) were used to evaluate the migration of HCC cells with or without MK (*n* = 10). All data are presented as mean ± SD. *P < 0.05, **p < 0.01, and ***p < 0.001 compared with the NC group, ^#^*P* < 0.05, ^##^*P* < 0.01, and ^###^*p* < 0.001 compared with the group without BKM or MK. *P*-value was calculated using Student’s *t*-test (**A**) and one-way analysis of variance with Tukey’s multiple comparison tests (**B**–**H**).

In this part, the mechanisms by which CD36 induces mTOR phosphorylation were studied. Early studies have found that CD36 physically interacts with Src kinases, likely leading to Src activation^23^. Therefore, we determined whether CD36 actives Src pathway in HCC. Consistent with previous findings, CD36 OE HCC cells had marked Src tyrosine kinase activation (Fig. 7A). Regarding the downstream effectors of Src, CD36 overexpression dramatically increased the phosphorylation of PI3K and also AKT (Fig. 7A). On the opposite, CD36 knockdown in SMMC-7721, HepG2, and PLC/PRF/5 cells largely restrained the Src/PI3K/AKT signaling pathway (Supplementary Fig. 2A–I).

Therefore, we want to determine whether CD36 affects the mTOR signal through PI3K/AKT pathway. Pharmacological inhibition of PI3K pathway with BKM-120 (1 μM) effectively blocked mTOR signal (Fig. 7B) and simultaneously prevented CD36-induced cell proliferation and migration (Fig. 7C–E). We also investigated whether the AKT inhibitor MK-2206 (5 μM) has the same effects. The results were that MK-2206 successfully inhibited cell proliferation and migration, and weakened the function of CD36 (Fig. 7F–H). All together, these data suggested CD36 activates the mTOR pathway through the Src/PI3K/AKT cascade.

## Discussion

The fatty acid receptor CD36 is emerging as a potential strategy for cancer treatment. The importance of CD36 in the regulation of proliferation, metastasis, and angiogenesis of different tumor types has been demonstrated, such as oral cancer, cervical cancer, gastric cancer, and leukemia. Elevated free fatty acid and CD36 levels associated with epithelial–mesenchymal transition in HCC patients have been reported, suggesting a potential but unrevealed role of CD36 in HCC^32^. In the present study, we found that the expression of CD36 is significantly elevated in HCC tissues and HCC cell lines. CD36 overexpression enhanced the proliferation, migration, and invasion of HCC cells in vitro and promotes HCC tumor growth and metastasis in vivo. In contrast, the ability of proliferation, migration, and invasion of HCC cells was suppressed with the disruption of endogenous CD36. Our data suggested that CD36 may participate in the pathogenesis of HCC, and it is worth noting that our findings may be the first to identify the regulatory role of CD36 in HCC.

CD36 is a central regulator for cell metabolism, maintaining lipid and glucose metabolism. The protein also transduces signaling to mediate its role in inflammation^33^, lipid metabolism, and insulin responsiveness, contributing to the pathogenesis of metabolic diseases, such as obesity, atherosclerosis, NAFLD, and type 2 diabetes^34^. The function of CD36 in fatty acid metabolism is well documented and several studies have revealed its association with cancer. CD36-mediated exogenous fatty acid uptake and oxidation leads to cancer growth and metastasis^23,34^. Metabolic reprogramming has been recognized as a hallmark of cancer, changes of glucose, fatty acid, and glutathione metabolic pathways are often involved. However, the most common metabolic change is the Warburg effect, in which cancer cells favor the use of less efficient aerobic glycolysis rather than mitochondrial oxidative phosphorylation, to metabolize glucose for cellular proliferation. This prompted us to think about whether CD36 may orchestrate glucose metabolism in tumor cells. There has been a growing interest in the possible role of CD36 in glucose metabolism. Type I CD36 deficiency rather than type II CD36 deficiency is associated with hypoglycemia in preschoolers^35^. Ours and others’ studies indicated that loss of CD36 impairs insulin signaling and glucose metabolism in the skeletal muscle and liver^16^. Herein, by analysis of HCC cell’s metabolic phenotype, we found CD36 expression is positively associated with ECAR, a measure of glycolysis, instead of OCR. Further experiments verified that upregulation of CD36 resulted in increased glycolysis flux and lactic acid production. Our study extended previous research by elucidating a fundamental role of in the regulation of glycolysis in HCC cells.

Recently, CD36 has been proposed as a new target for antitumor therapy. Drugs targeting CD36 have already entered in clinical trials, but many candidates failed due to adverse effects and unsatisfactory performance^36^. Hence, there is a pressing need to further investigate the downstream targets of CD36. In the present work, we focus on the downstream mediators of CD36 to induced glycolysis in HCC. CD36 is a multi-functional protein that participates in a variety of signal transduction pathways^37^. However, very little is known about the molecular mechanisms by which CD36 mediates carcinogenesis. Studies have indicated that the mTOR pathway is a key regulator of glycolysis and plays an important role in the growth, survival, and migration of cancer cells^38,39^. Then, we asked that whether CD36 mediates glycolysis through mTOR pathway. Our results showed that mTOR signaling pathway could be activated by CD36 in HCC and inhibition of mTOR eliminates CD36-mediated glycolysis and tumor-promoting effects. Thus, mTOR is a novel downstream target of CD36-mediated glycolysis in HCC.

CD36-mediated intracellular signals could be initiated by the physical association of the Src protein tyrosine kinase, which is regarded as an oncogene in the development of cancer. Our previous work has also demonstrated that upregulation of Src pathway by CD36 is a potential mechanism to induce cervical cancer cell growth and metastasis^23^. To further explore the potential mechanism of how CD36 affects mTOR phosphorylation, we investigated whether CD36 mediated mTOR through Src kinase and its downstream effectors. Interestingly, CD36 overexpression not only increased the phosphorylation of Src but also activated its key downstream targets, namely PI3K and AKT. PI3K/AKT is an upstream regulator of mTOR, which is also crucial for the regulation of glycolysis by inducing glycolytic enzymes^40,41^. Thus, our results suggested that CD36 dramatically activates mTOR phosphorylation via Src/PI3K/AKT signal axis, resulting in enhanced glycolytic pathway, and ultimately promotes HCC growth and metastasis.

In conclusion, our findings demonstrate that (1) CD36 is highly expressed in HCC and elevated CD36 expression contributes to HCC tumor growth and metastasis; (2) CD36 plays a fundamental role in the metabolic reprogramming of glycolysis in HCC cells; and (3) CD36 induces mTOR, which is mediated by the upregulation of Src/PI3K/AKT signal axis. Altogether, our study suggests that CD36, by activating glycolysis through Src/PI3K/AKT/ mTOR signaling pathway, could be a critical step in the development and progression of HCC; thus, CD36 may be a novel target for liver cancer therapy.

## Supplementary information

Supplement Figure 1

Supplement Figure 2
